# Kidney transplantation between identical twins with APOL1 homozygote risk alleles – a case report

**DOI:** 10.3389/fneph.2025.1648263

**Published:** 2025-07-31

**Authors:** Sean Lei, Abhirami Shankar, Supreet Sethi, Erik L. Lum

**Affiliations:** ^1^ Division of Nephrology, Department of Medicine, David Geffen School of Medicine - University of California, Los Angeles, CA, United States; ^2^ Division of Nephrology, Harbor University of California Los Angeles Medical Center, Los Angeles, CA, United States

**Keywords:** kidney transplant, kidney transplantation, living donor, APO L1, genetic kidney disease

## Abstract

Kidney transplantation is the optimal therapy for individuals with end-stage kidney disease. Recent studies suggest a negative impact of high-risk Apolipoprotein L1 genotypes on outcomes for both living kidney donors and kidney transplant recipients. In this case, we describe a pair of identical twins with a high-risk APOL1 genotype who underwent successful living kidney transplantation with excellent short-term outcomes.

## Introduction

Kidney transplantation is the optimal therapy for individuals with end-stage kidney disease. Recent studies suggest a negative impact of high-risk Apolipoprotein L1 genotypes on outcomes for both living kidney donors and kidney transplant recipients. In this case, we describe a pair of identical twins with a high-risk APOL1 genotype who underwent successful living kidney transplantation with excellent short-term outcomes.

## Case presentation

A black female in her late 40s presented for kidney transplant evaluation. She had a longstanding history of hypertension dating back to her early 20s and had been on dialysis for two years prior to evaluation. A kidney biopsy performed several years earlier demonstrated focal segmental glomerulosclerosis (FSGS). Her transplant evaluation was unremarkable and she was placed on the kidney transplant waiting list. After six years of waiting, her twin sister came forward as a potential donor. The patient was in her late-50s at the time. Given the recipient’s young age at onset of kidney disease, race and for prognostication after kidney transplantation, genetic testing was performed, revealing high-risk APOL1 genotype (homozygote G1).

The donor was confirmed to be an identical twin using single nucleotide repeat testing and carried the same high-risk APOL1 genotype. Her medical evaluation was unremarkable, with a serum creatinine of 0.7 mg/dL, a serum cystatin C of 0.9 mg/dL, with a combined Cr-Cystatin-C eGFR of 95 mL/min/1.73m^2^ and no proteinuria. Additional testing with a 24-hour urine collection showed a normalized creatinine clearance of 124 mL/min/1.73m2, urine protein <4 mg/dL, and urine albumin <12 mg/L. She was normotensive at the time of evaluation, and a 24-hour ambulatory blood pressure monitor recorded an average BP of 126/73 mmHg. The potential risk of post-donation chronic kidney disease (CKD)/end stage kidney disease (ESKD) was discussed in detail with the donor especially in the context of her high risk APOL1 genotype. However, given her relatively older age with no signs of kidney disease into her sixth decade of life, and normotension, she was allowed to proceed as a donor after obtaining an informed consent.

**Figure 1:**
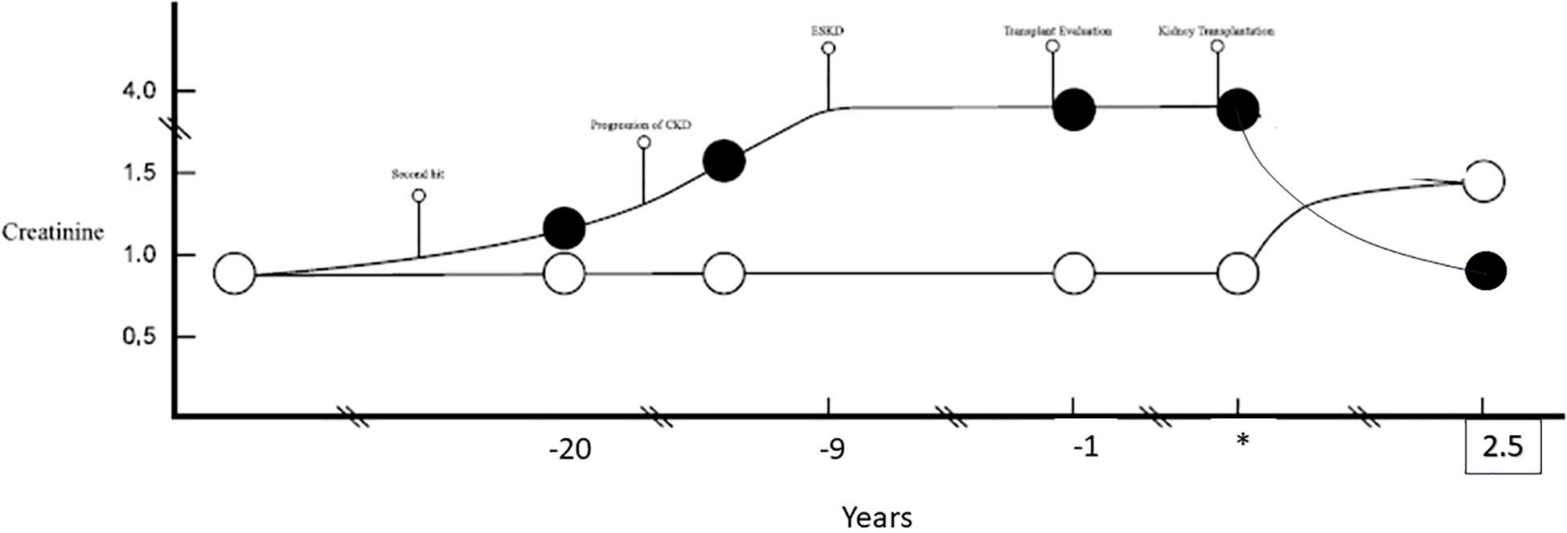
A chronological presentation of two identical twins based on the respective kidney functions (serum creatinine). The recipient (black circle) likely experienced progressive CKD with progression to ESKD. *Marks the time of kidney transplantation and time points are reflected in years prior (negative) and post (positive) kidney transplant. The donor (white circle) remained with unchanged creatinine. Post kidney transplantation the donor and recipient have maintained steady creatinine for over 2.5 years post living donor transplantation.

The pair underwent successful kidney transplantation without immunosuppression. The recipient is now two and a half years post kidney transplant without immunosuppressive therapy, and has a stable kidney allograft function, with a serum creatinine of 0.9 mg/dL (eGFR of 70 mL/min/1.73m^2^)and no proteinuria. Post-donation, the donor experienced significant weight gain, with her BMI increasing from 29 to 41. Otherwise, her renal function has remained stable, with a serum creatinine of 1.24 mg/dL (eGFR of 50 mL/min/1.73m^2)^, urine protein-creatinine ratio of 0.1, and home-recorded blood pressures averaging 130/80 mmHg (**Figure 1**).

## Discussion

We present a case of identical twin siblings in their late 50s with a high risk APOL1 genotype undergoing living donor kidney transplantation. In this case, the potential risks associated with the high-risk APOL1 genotype in both individuals were carefully assessed, and with shared decision making and informed consent, transplantation proceeded, resulting in excellent short-term outcomes for both the donor and recipient, whom is immunosuppression free for two years.

The role of genetic diseases and risk variants has garnered increased significance in the evaluation of patients with CKD. It is estimated that 30–50% of CKD cases in children, and 10–20% in adults, are caused by distinct monogenic disorders (1). Identification of genetic variants aids in the prognosis and management of patients with CKD. Recent technological advancements have reduced the costs of genetic testing in patients with CKD, and recently KDOQI updated their guidelines to recommend genetic testing in patients with CKD and potential kidney donors who have a strong family history of CKD or a family member with a known pathogenic genetic variant (2).

However, widespread genetic testing in potential donors and patients with CKD results in the identification of genetic variants of uncertain significant and absence of phenotypical disease despite the presence of pathogenic gene variants as not all genetic variants are fully penetrant. This can complicate patient counseling and pose challenges in risk assessment and clinical decision-making for potential living donors.

APOL1 is a component of high-density lipoprotein (HDL), encoded on chromosome 22 (3). Approximately 35% of African Americans carry a high-risk APOL1 genetic variant (G1 or G2), and 13% carry two risk alleles (either G1/G1, G2/G2, or G1/G2) (4, 5). The presence of two risk alleles, known as a high-risk genotype, is associated with an increased risk of non-diabetic proteinuric CKD and kidney disease progression. However, heterozygous individuals, who carry a wild-type allele (G0) along with a single copy of a high-risk allele, are not at increased risk for kidney disease (5). Despite its well-established association with kidney disease, high-risk genotype only increases an individual’s lifetime risk of kidney disease by approximately 15%, and the majority of individuals with this genotype do not develop kidney disease (3). This variability in phenotypic expression is thought to be explained by the “two-hit hypothesis,” which suggests that, in addition to genetic predisposition, an environmental or secondary factor is required to trigger disease manifestation (6). This phenomenon is evident in our case, where one identical twin developed ESKD in her 40s, while the donor had no evidence of kidney disease well into her sixth decade of life with an extensive work up demonstrating excellent kidney function without proteinuria based on a 24-hour urine collection and estimated GFR from both creatinine and cystatin-C equations.

The presence of a high-risk APOL1 genotype has been associated with an increased risk of post-donation CKD. In a study by Doshi et al., 19 patients with a high-risk genotypes were identified, two of whom developed ESKD following donation (7). At 12 years post-donation, donors with a high-risk APOL1 genotype had a significantly lower GFR (57 vs. 67 mL/min) and a faster eGFR decline compared to 117 low-risk genotype donors. However, the small sample size of high-risk genotype donors, the young average age of donors (37 years), and the reduced renal function at the time of donation in the high-risk genotype group limit the generalizability of these results.

Absence of phenotypic kidney disease in the presence of a pathogenic genotype may play a critical role in determining future CKD risk in individuals with high-risk APOL1 genotypes as demonstrated in another study by Doshi et al. (8). In this study, using data from the Atherosclerosis Risk in Communities (ARIC) Study, healthy Black individuals aged 45–64 years with high-risk APOL1 genotypes and an eGFR >80 mL/min showed no additional risk for developing ESKD, proteinuria, or CKD 3a and higher compared to the low-risk APOL1 group over 25 years of follow-up (8). This study suggests that individuals who reach middle age without evidence of kidney disease have a low lifetime risk of developing CKD. Moreover, after age 60, similar long term outcomes exist in European American and African American living kidney donors (9). In genetic conditions with incomplete penetrance, such as APOL1, donor age and future risk for CKD should be carefully considered when determining donor safety. Given our donor’s age and the absence of detectable kidney disease, her lifetime risk of CKD was considered low, and she was cleared to donate.

The presence of high risk APOL1 genotypes in the donated kidney is associated with worse post-transplant outcomes, especially if the recipient also carries a high risk APOL1 genotype. This risk may not be limited to just the short term. In another case of an identical twin kidney transplant, which occurred in their 30s, the recipient developed proteinuria of 1.8 gm 18 years post transplantation (10). A kidney biopsy demonstrated patchy interstitial fibrosis and FSGS. APO L1 testing at time of biopsy in both the donor and recipient demonstrates G1/G2 high risk APO L1 variant. The authors argue that late recurrence can occur. However, the donor developed no evidence of kidney disease after 18 years, demonstrating long term donor safety and one could argue that 18 years without immunosuppression and ongoing kidney function in the recipient is a far superior transplant outcome. The long term effects of APO L1 genetic variants in kidney transplantation is being studied prospectively in the ongoing NIH funded APOLLO (APOL1 Long term Kidney Transplantation Outcomes Network) trial. In addition, the retrospective LETO (Living-donor Extended Time Outcomes) study analyzing large numbers of African American living donors will provide critical information on the safety of living kidney donation).

While there is an associated risk of worse allograft function, this must be considered (11). In the case described above, the risk for inferior graft survival was weighed against the benefit of living kidney transplant, morbidity and mortality associated with remaining on dialysis while waiting for a deceased donor kidney, and the ability to avoid long term immunosuppression (11–15). We acknowledge the current limited follow up of our donor-recipient pair and the need to continue to monitor them closely in the long term. Of note, donor is actively working on losing weight.

## Conclusion

The increasing utilization of genetic testing for kidney transplant donors may identify potential donors at increased risk of post donation kidney disease. However, the presence of high risk genetic variance in the absence of phenotypic evidence of kidney disease in middle aged donors should not alone result in decline. In this case, after careful consideration of the risks and benefits, a pair of identical twins with high risk APOL1 genotypes underwent living donor kidney transplant with excellent short term outcomes for both the donor and recipient.

## Data Availability

The raw data supporting the conclusions of this article will be made available by the authors, without undue reservation.
